# Heat stress in pigs and broilers: role of gut dysbiosis in the impairment of the gut-liver axis and restoration of these effects by probiotics, prebiotics and synbiotics

**DOI:** 10.1186/s40104-022-00783-3

**Published:** 2022-11-18

**Authors:** Robert Ringseis, Klaus Eder

**Affiliations:** Institute of Animal Nutrition and Nutrition Physiology, Justus-Liebig-University Giessen, Heinrich-Buff-Ring 26-32, 35392 Giessen, Germany

**Keywords:** Broilers, Commensal bacteria, Gut dysbiosis, Heat stress, Pigs, Prebiotics, Probiotics, Synbiotics

## Abstract

Heat stress is one of the most challenging stressors for animal production due to high economic losses resulting from impaired animal’s productivity, health and welfare. Despite the fact that all farm animal species are susceptible to heat stress, birds and pigs are particularly sensitive to heat stress due to either lacking or non-functional sweat glands. Convincing evidence in the literature exists that gut dysbiosis, a term used to describe a perturbation of commensal gut microbiota, develops in broilers and pigs under heat stress. Owing to the protective role of commensal bacteria for the gut barrier, gut dysbiosis causes a disruption of the gut barrier leading to endotoxemia, which contributes to the typical characteristics of heat stressed broilers and growing and growing-finishing pigs, such as reduced feed intake, decreased growth and reduced lean carcass weight. A substantial number of studies have shown that feeding of probiotics, prebiotics and synbiotics is an efficacious strategy to protect broilers from heat stress-induced gut barrier disruption through altering the gut microbiota and promoting all decisive structural, biochemical, and immunological elements of the intestinal barrier. In most of the available studies in heat stressed broilers, the alterations of gut microbiota and improvements of gut barrier function induced by feeding of either probiotics, prebiotics or synbiotics were accompanied by an improved productivity, health and/or welfare when compared to non-supplemented broilers exposed to heat stress. These findings indicate that the restoration of gut homeostasis and function is a key target for dietary interventions aiming to provide at least partial protection of broilers from the detrimental impact of heat stress conditions. Despite the fact that the number of studies dealing with the same feeding strategy in heat stressed pigs is limited, the available few studies suggest that feeding of probiotics might also be a suitable approach to enhance productivity, health and welfare in pigs kept under heat stress conditions.

## Introduction

Among several stressors affecting animal production, heat stress is considered as one of the most challenging ones due to the high annual economic losses resulting from decreased animal productivity (e.g. reduced feed intake, decreased growth rate, impaired reproduction, reduced lactation performance) and welfare and increased morbidity and mortality [1, 2]. According to estimations for the United States livestock industry, the annual economic losses resulting from heat stress amount to 1.5 and 1 billion dollars for dairy cattle and pigs, respectively [3, 4]. Owing to the ongoing increase of global temperature [5], and the increasing frequency of extreme heat events (e.g. prolonged phases of hot days) observed in many regions of the world [6, 7], heat stress has become a significant problem for animal production not only in tropical regions but also in temperate climate zones. Heat stress occurs when the ambient temperature exceeds the animal’s thermoneutral zone and the physiological capacity to dissipate heat via sweating, respiration or panting fails to prevent an increase of body temperature [8]. Despite that all farm animal species are susceptible to heat stress, birds and pigs are particularly sensitive to heat stress due to either lacking or non-functional sweat glands [9, 10]. Since the animal’s thermoneutral zone decreases during growth, growing farm animals, such as broilers and growing-finishing pigs, are more susceptible to heat stress during the finishing period than during the starter period. Remarkably, the high susceptibility of growing farm animals used for meat production to heat stress has been aggravated by the fact that modern breeds are the result of intense genetic selection for enhanced lean carcass growth (muscle growth) [11], which is accompanied by an increased basal heat production. Thus, the ongoing climate change in combination with the use of high-production phenotypes is going to promote problems associated with heat stress in animal production in the future, thus, increasing the need to develop strategies to combat heat stress.

**Fig. 1:**
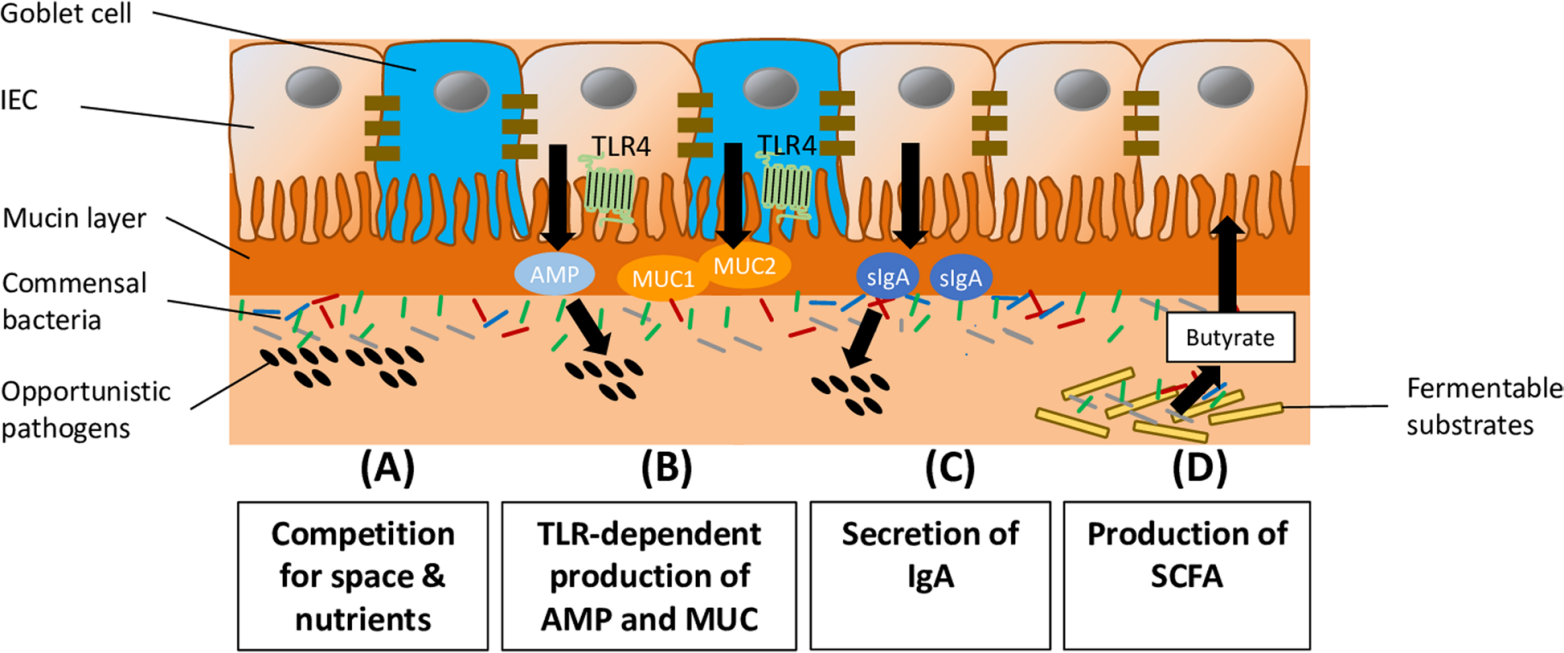
Important mechanisms of commensals involved in colonization resistance. The commensals in the gut play a key role in preventing the expansion of potential pathogens, thereby, contributing to colonization resistance. The most important mechanisms of commensals involved in colonization resistance comprise: (**A**) Commensals provide a direct barrier to colonization by pathogens through competing for space and nutrients; (**B**) Commensals continuously stimulate pathogen recognition receptors (PRR), like Toll-like receptors (TLR), on intestinal epithelial cells (IEC) to secrete protective mucins (MUC) and antimicrobial peptides (AMP); (**C**) Commensals contribute to adaptive immunity by stimulating secretion of secretory immunoglobulin A (IgA) which provides protection against pathogens by crosslinking of pathogenic bacteria and neutralizing bacterial toxins; (**D**) Commensals generate microbial fermentation products, such as butyrate, which has trophic effects on the intestinal mucosa, thereby, enhancing the intestinal barrier

While heat stress is known to cause a wide array of pathophysiological changes, the organ which is particularly sensitive to heat stress is the gut and the disruption of the gut barrier, a critical element of the gut-liver axis, has been recognized as the starting point of the negative consequences on animal productivity, welfare and health [12]. Despite that hypoperfusion of the gut is widely considered as the key event in the development of gut barrier disruption during heat stress, gut dysbiosis, which has been recently documented in different farm animal species including broilers and pigs exposed to heat stress [13, 14], might also contribute to gut barrier disruption during heat stress. In fact, the commensal bacteria in the gut play an outstanding role in strengthening the gut barrier via different mechanisms [15]. These beneficial mechanisms of the commensals involved in colonization resistance comprise the provision of a direct barrier to colonization by pathogens through competing for space and nutrients [16]. In addition, commensals continuously stimulate pathogen recognition receptors (PRR), like Toll-like receptors (TLR), on intestinal epithelial cells (IEC) to secrete protective mucins and antimicrobial peptides [17]. Moreover, commensals contribute to adaptive immunity by stimulating secretion of secretory immunoglobulin A (IgA) [18]. Furthermore, commensals generate microbial fermentation products, such as butyrate, which has trophic effects on the intestinal mucosa, thereby, enhancing the intestinal barrier [19]. The most important mechanisms of commensals involved in colonization resistance are summarized in Fig. 1. Accordingly, a perturbation of commensal gut microbiota, which is referred to as gut dysbiosis, causes a weakening of the gut barrier and results in intestinal hyperpermeability and an increased translocation of bacterial endotoxins, such as lipopolysaccharide (LPS), into the circulation [20], a condition called endotoxemia. Endotoxemia in turn causes a dysregulation of intermediary metabolism and a reduction of feed intake, both of which are important characteristics of heat stressed animals, through inducing local and systemic inflammation. Because gut dysbiosis impairs all decisive structural, biochemical and immunological elements of the intestinal barrier, dietary strategies aiming to restore a normal gut microbiota are likely to protect the animal from heat stress-induced gut barrier disruption and metabolic derangements. Dietary strategies which have been demonstrated to be effective in combating gut dysbiosis in pigs and broilers include the feeding of probiotics, prebiotics and synbiotics [21, 22]. Based on this, the present review aims to 1) provide current evidence on the occurrence of gut dysbiosis and the possible role of gut dysbiosis in the development of gut barrier disruption and feed intake reduction in heat stressed broilers and pigs, and 2) comprehensively evaluate the efficacy of supplementation with probiotics, prebiotics and synbiotics on gut barrier function and performance in broilers and pigs exposed to heat stress.

## Heat stress impairs the intestinal barrier as a key structural element of the gut-liver axis in broilers and pigs

A very early response of the body to heat stress is vasoconstriction in the splanchnic circulation, which supplies the gastrointestinal tract, liver and other visceral organs with blood, with a consequent shift of the blood to the periphery in order to dissipate heat via the skin and to decrease body core temperature [23]. As a result of this, hypoxia and a decrease of nutrient delivery occurs in the intestinal tissue, which is considered to be mainly causative for an impairment of intestinal integrity and disruption of intestinal barrier under heat stress [24, 25]. The intestinal barrier is a critical structural element of the gut-liver axis serving as a physical and functional barrier between the intestinal microbiota and the liver, facilitating absorption of nutrients, while hindering the translocation of commensal and pathogenic microorganisms [26]. The intestinal barrier is largely based on a single layer of IEC, which are tightly connected by intercellular junctions sealing the space between adjacent cells and controlling paracellular passage, and a mucin layer, which consists of an inner, dense and an outer, loose layer and covers the luminal surface of the IEC. In addition, the commensal bacteria closely associated with the mucin layer and several immunologic molecules, such as antimicrobial peptides and secretory IgA, derived from IEC and a network of innate and adaptive immune cells located in the lamina propria, respectively, are vital to intestinal barrier function.

A large body of evidence exists that heat stress impairs almost all elements of the intestinal barrier. Studies in broilers and pigs showed that the number of goblet cells – a subtype of IEC specialized for the production of highly glycosylated proteins called mucins (MUC), such as MUC2 (the most abundant), MUC1 and others – is reduced under heat stress [27, 28]. In line with this function of goblet cells, the expression and secretion of mucins was found to be decreased in broilers and pigs exposed to heat stress [27], which causes thinning of the protective mucin layer. The important role of the glycoproteins of the mucin layer, which is important in preventing the direct bacterial contact with the IEC, is demonstrated by the observation that expression of the predominant mucins contributes to resistance against opportunistic intestinal pathogens, such as *Campylobacter jejuni* and *Salmonella enterica* serovar Typhimurium [29, 30]. Besides, the mucin layer is also important for commensal bacteria colonizing the outer mucin layer, because its polysaccharide content serves as a nutrient source for bacterial fermentation, thereby, contributing to intestinal integrity and anti-inflammatory actions.

Importantly, the commensal gut bacteria, which symbiotically colonize the animal’s intestine, strongly influence IEC physiology through a variety of pattern recognition receptors, such as TLR, which recognize and bind specific microbial ligands and are important in developing immune tolerance against harmless antigens and commensal bacteria and maintaining homeostasis of the intestinal barrier. In contrast, a perturbation of commensal gut microbiota induces an activation of the gut-associated lymphoid tissue consisting of intraepithelial lymphocytes, Peyer’s patches and a network of immune cells in the lamina propria, an inflammatory response of the IEC and a disruption of the epithelial barrier. To a large extent, the beneficial role of commensal bacteria on intestinal barrier integrity is explained by their stimulatory effect on mucin production by goblet cells, which is mediated in a TLR/myeloid differentiation primary response 88 (MyD88)-dependent manner [31]. MyD88 is a cytosolic adaptor protein converting extracellular signals recognized by TLR to intracellular responses, such as nuclear factor-κB (NF-κB) activation and cytokine production. In addition, commensal bacteria stimulate Paneth cells – highly specialized secretory IEC located in the small intestinal crypts – to express antimicrobial peptides, such as α-defensins, β-defensins, cathelicidins and C-type lectins, like regenerating islet-derived 3γ [32]. The antimicrobial peptides, which are also secreted from enterocytes – the predominant type of IEC, are not only important for limiting colonization of the inner mucin layer by pathogenic microbes, but also for controlling the growth of commensal gut bacteria [33]. Apart from antimicrobial peptides, secretory IgA plays a central role in the defense against intestinal pathogens at the luminal mucosa surface by crosslinking of pathogenic bacteria and neutralizing bacterial toxins [18]. Despite that IgA is produced from IgA-producing plasma cells in the intestinal lamina propria, the IgA is ultimately secreted from IEC into the intestinal lumen. In order to fulfil this function, IEC express a specific receptor on the basolateral side of IEC. This receptor mediates the uptake and transcytosis of IgA to the apical surface from which it is secreted into the intestinal lumen. Moreover, commensal bacteria contribute to intestinal barrier integrity through modulation of the gut immune system. For instance, commensal bacteria were shown to induce development and maturation of intestinal lymphoid structures, such as Peyer´s patches and isolated lymphoid follicles [34, 35]. Furthermore, the protective effect of commensal bacteria on the gut barrier is explained by the fact that commensal bacteria prevents colonization by pathogenic bacteria through competing for space and nutrients [15]. Finally, several commensal bacteria produce short-chain fatty acids (SCFA), such as butyrate, from fermentation of indigestible carbohydrates; butyrate has long been known for its trophic effects on IEC [36], thereby, contributing to gut barrier integrity.

## Intestinal barrier disruption by heat stress causes intestinal hyperpermeability and endotoxemia

Increased bacterial penetration of the inner mucin layer has been shown to induce IEC inflammation and increased apoptosis and shedding of IEC at the villus tip. As a mechanism of action, TLR4-dependent sensing of microbial-associated molecular patterns (MAMP), like LPS, with the consequence of gap formation in the epithelium, permeability defects and villus shortening (villus atrophy) has been identified [37, 38]. In agreement with the negative impact of heat stress on determinants of the protective mucin layer in broilers and pigs, heat stressed broilers and pigs were demonstrated to develop intestinal mucosa inflammation, as evident from inflammatory cell infiltration, lamina propria edema and pathological intestinal epithelial shedding [14, 39]. In addition, intestinal inflammation was accompanied by intestinal morphological changes being characteristic of intestinal damage, such as villus shortening and decrease of villus height-to-crypt depth ratio in heat stressed broilers and pigs [40, 41]. Rapid and uncoordinated shedding of IEC results in the disruption of paracellular tight junctions, which not only serve to link adjacent IEC to each other, but are also critical regulators of epithelial cell proliferation, differentiation and polarization [42]. Typically, disruption of tight junctions is accompanied by relocalization and altered expression of tight junction protein constituents, such as occludin (OCLN), various claudins (CLDN) and zonula occludens (ZO)-1, -2 and -3. In line with the detrimental impact of heat stress on the intestinal barrier, short-term and long-term exposure of broilers and pigs to heat stress was also found to cause an altered expression and localization of different tight junction proteins being indicative of a deterioration of gut barrier function [28, 43, 44]. Interestingly, intestinal tight junction proteins are differentially regulated by bacterial stimulation of specific TLR. Stimulation of TLR4 by LPS from Gram-negative bacteria was shown to decrease barrier function by TLR4/MyD88-dependent upregulation of myosin light chain kinase, which induces opening of tight junctions [45]. In contrast, activation of TLR2 by Gram-positive gut commensals, such as *Lactobacillus* spp. including *L. acidophilus* and *L. plantarum*, which are frequently used as probiotics, results in increased occludin protein expression and apical relocalization of ZO1, thereby, improving barrier function [46, 47]. Likewise, the probiotic *Bifidibacterium infantis* was shown to improve intestinal barrier function by preserving CLDN4 and OCLN localization along the vicinity of the tight junctions [48]. These findings clearly show that specific TLR play distinct roles in the regulation of intestinal tight junctions and that an imbalance between commensal- and pathogen-induced activation of TLR resulting from gut dysbiosis impairs gut homeostasis and barrier function. In this regard, oral administration of Gram-positive gut commensals, such as *Lactobacillus* spp. and *Bifidobacterium* spp., both of which are important sources of TLR2-stimulating ligands [49, 50] is a reasonable strategy to improve barrier function by promoting beneficial effects on intestinal tight junctions.

As a consequence of heat stress-induced intestinal barrier dysfunction, the resulting intestinal hyperpermeability, which has been demonstrated in heat stressed pigs and broilers [51–54], causes translocation of MAMP, such as LPS and bacterial DNA, and even intact microbes into the portal circulation. Owing to the ability of the liver to capture and/or kill whole bacteria and MAMP by liver-resident macrophages (Kupffer cells) and several other immune cells, which are equipped with specific PRR to sense and respond to MAMP [55], an increased translocation of MAMP into the systemic circulation is normally prevented, thus, protecting other tissues from inflammatory stimuli. However, if the intestinal barrier is severely disrupted (“leaky gut”), the capacity of the liver to cope with high levels of MAMP will be exceeded leading to endotoxemia and local and systemic inflammation.

## Endotoxemia during heat stress causes dysregulation of intermediary metabolism and feed intake by inducing hepatic and hypothalamic inflammation

Convincing evidence exists that endotoxemia resulting from loss of intestinal barrier integrity causes a dysregulation of feed intake and intermediary metabolism [56], with key events being hepatic and hypothalamic inflammation. As a consequence of the activation of hepatic PRR, such as TLR and nucleotide-binding and oligomerization domain-like receptors, proinflammatory cytokines [IL1β, tumor necrosis factor α (TNFα)] and chemokines are produced and released. Both, cytokines and chemokines cause a recruitment of further inflammatory cells, thereby, inducing a local inflammatory response in the liver, called hepatic inflammation. Because the liver-derived proinflammatory mediators are also secreted into the circulation, systemic inflammation develops, which also affects peripheral tissues, like skeletal muscle. In agreement with this, elevated levels of proinflammatory mediators and LPS in the blood of pigs and broilers exposed to heat stress are well-documented [52, 53]. In the skeletal muscle, proinflammatory cytokines and LPS are well-known to decrease protein synthesis, while stimulating protein catabolism [57–59]. In line with this, protein deposition is reduced and blood markers of muscle protein degradation, such as creatine, creatinine and 3-methylhistidine are increased in pigs and broilers under heat stress [51, 60]. In addition, skeletal muscle becomes insulin-resistant during heat stress, thereby, decreasing glucose utilization by skeletal muscle. This provides an important mechanism shifting the glucose away from skeletal muscle to the site of the immune response [12]. Interestingly, despite that feed intake typically decreases during heat stress, lipid carcass deposition is increased in growing pigs and broilers under heat stress as a result of reduced lipid mobilization and increased lipogenesis in white adipose tissue [61–63]. This effect is likely mediated by the elevated insulin levels repeatedly observed in a variety of heat stressed animal species including pigs and broilers [63, 64], because insulin is a stimulator of lipogenesis and an inhibitor of lipolysis. A further striking metabolic alteration in heat stressed monogastric farm animals is an increased intestinal glucose uptake and elevated hepatic glucose output. These processes are supposed to enable heat stressed animals to enhance whole-body utilization of glucose during heat stress [12]. The metabolic changes occurring in heat stressed growing pigs and broilers likely serve to prioritize the local and systemic immune response arising from intestinal barrier disruption, while muscle protein accretion and body weight gain are attenuated. The main reason for this is that the immune response requires an increased supply of glucose and amino acids as preferential energy substrates and building blocks for the synthesis of acute phase proteins and antibodies, respectively.

**Fig. 2:**
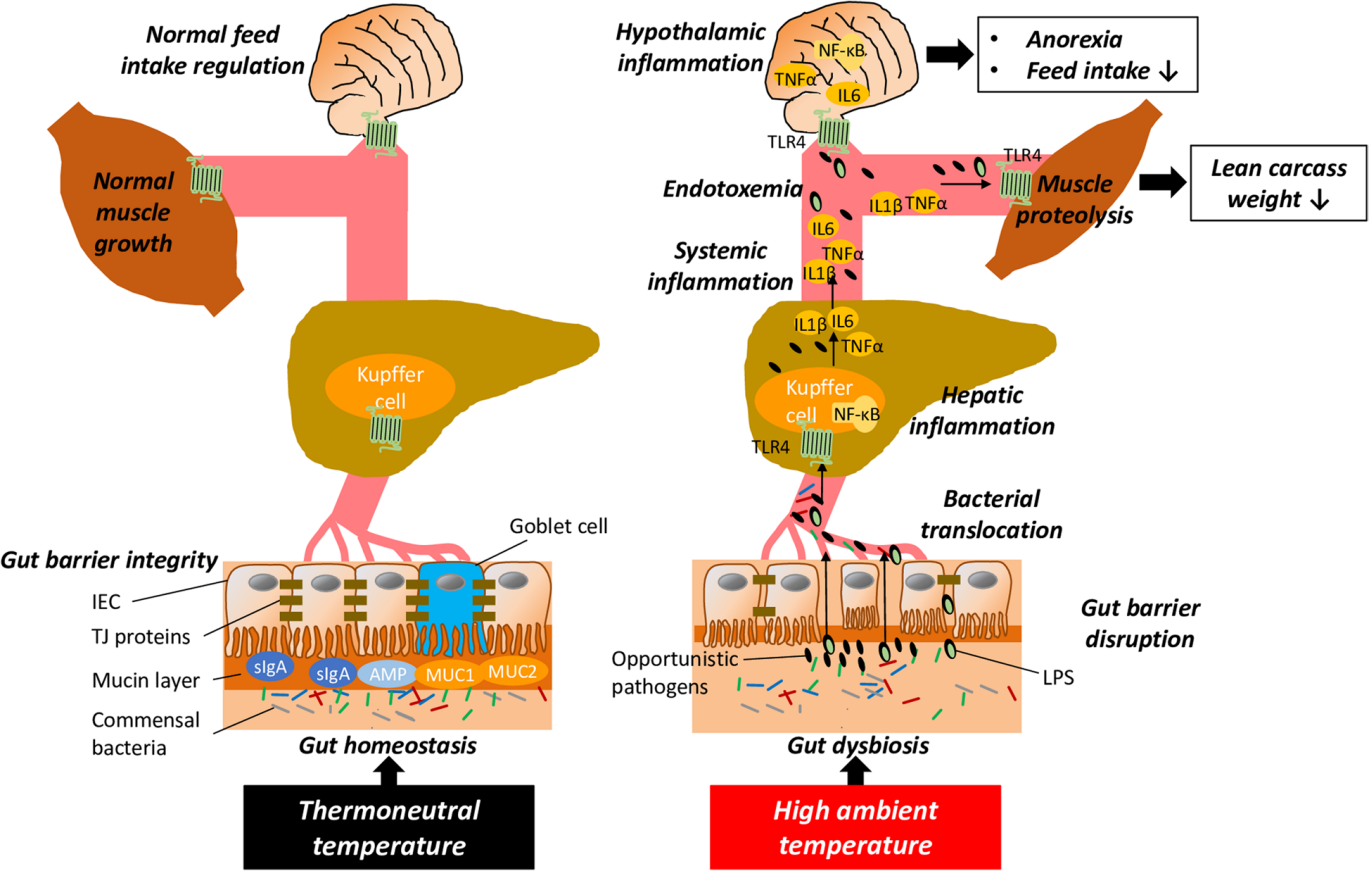
Role of gut dysbiosis in the development of gut barrier disruption, endotoxemia and hepatic and hypothalamic inflammation under heat stress. A large body of evidence exists that gut dysbiosis, a term used to describe a perturbation of commensal gut bacteria community, develops in broilers and pigs kept at high ambient temperature exceeding the thermoneutral zone. At thermoneutral zone, commensal bacteria promote gut barrier integrity through inhibiting the colonization by pathogens, continuously stimulating intestinal epithelial cells (IEC) to secrete protective mucins (MUC1, MUC2) and antimicrobial peptides (AMP) and contributing to adaptive immunity by stimulating secretion of secretory immunoglobulin A (sIgA). In contrast, at high ambient temperature gut dysbiosis is occurring which leads to an overgrowth of opportunistic pathogens, thereby, leading to a weakening of all decisive structural, biochemical, and immunological elements of the gut barrier, which is visible by a decreased expression and relocalization of tight junction (TJ) proteins, loss of goblet cells, reduced production of mucins, thinning of mucin layer, IEC shedding and IEC villus shortening and hyperpermeability of the gut barrier. As a consequence, intact bacteria and bacterial components, such as lipopolysaccharide (LPS), translocate into the portal vein and cause hepatic inflammation via Toll-like receptor 4 (TLR4)-dependent activation of nuclear factor-kappa B (NF-κB). Activation of NF-κB stimulates production of pro-inflammatory cytokines, such as interleukin (IL1β), IL6 and tumor necrosis factor α (TNFα), which together with LPS enter the systemic circulation, thereby, causing endotoxemia and systemic inflammation. While elevated levels of proinflammatory cytokines and LPS stimulate muscle proteolysis and inhibit muscle protein synthesis, thereby, decreasing lean carcass growth, these cytokines and LPS cause TLR4-dependent hypothalamic inflammation. Hypothalamic inflammation causes activation of neuronal populations that produce anorexigenic neuropeptides, while inhibiting hypothalamic neurons expressing orexigenic neuropeptides, thereby, causing anorexia and decreasing feed intake, which are typical characteristics of broilers and pigs kept under heat stress conditions

Apart from hepatic inflammation, hypothalamic inflammation is known to develop in response to endotoxemia caused by endothelial barrier disruption. Hepatic inflammation, which arises from activation of hypothalamic PRR, particularly TLR4, by the systemically elevated levels of proinflammatory cytokines and MAMP [65], likely plays a fundamental role in the reduction of feed intake under heat stress. Activation of hypothalamic TLR4 and other PRR stimulates multiple inflammatory signaling pathways including NF-κB. As a consequence, a paracrine inflammatory milieu is induced in the hypothalamus, which alters the activity of neuronal populations regulating appetite and feed intake; specifically, acute hypothalamic inflammation causes activation of neuronal populations that produce anorexigenic neuropeptides [proopiomelanocortin (POMC), cocaine- and amphetamine-regulated transcript (CART)], while inhibiting hypothalamic neurons expressing orexigenic neuropeptides [agouti-related peptide (AgRP), neuropeptide Y (NPY)] [66]. As a result, appetite and feed intake are decreased. This mechanism explains why intravenous administration of LPS and proinflammatory cytokines in livestock animals including broilers and pigs strongly reduces feed intake [67–69]. Activation of NF-κB and elevated expression of various proinflammatory cytokines, such as IL6, IL18 and TNFα, in the hypothalamus has been recently documented in laying hens exposed to cyclic heat stress for 21 d [70]. In addition, several studies in avian species including broilers have demonstrated an altered expression of hypothalamic neuropeptides or peripheral signals involved in feed intake regulation, such as neuropeptide Y and ghrelin, under conditions of heat stress [71–74]. This indicates that heat stress affects central feed intake regulation, likely, as a consequence of hepatic inflammation. Acute hypothalamic inflammation is centrally involved in initiating the acute illness response, which represents an orchestrated response to infectious stimuli aiming to promote survival. Typical symptoms of the acute illness response, such as anorexia and cachexia, are clearly indicative of dysregulation of appetite and energy homeostasis by hypothalamic inflammation. In Fig. 2, the pathophysiological effects of heat stress causing anorexia, feed intake reduction and decrease of lean carcass weight are schematically summarized.

## Contribution of gut dysbiosis to the development of gut barrier disruption and feed intake reduction under heat stress

Apart from heat stress-induced hypoperfusion of the gut and the resulting hypoxia in intestinal mucosal tissue, which contribute to gut barrier disruption, heat stress was found to cause significant alterations of the gut microbiota in various species including broilers and pigs (see below). In recent years, it has become increasingly clear that the gut microbiota profoundly affects gut barrier function, feeding behavior and energy metabolism of the host [75]. This is impressively demonstrated by studies showing that the transfer of an “obesogenic” gut microbiota from an obese animal to a healthy lean animal by feces transplantation causes gut barrier disruption and induces metabolic disturbances, such as obesity, diabetes mellitus and metabolic syndrome [76, 77]. This profound influence of the gut microbiota on the animal’s metabolism is explained by the ability of the gut microbiota to communicate with the host along the gut-liver axis. Microbiota-host communication is mediated by different gut-derived compounds including SCFA and MAMP which are sensed by multiple host receptors, thereby, stimulating signaling and metabolic pathways in all key tissues of energy metabolism and feed intake regulation.

### Evidence for the development of gut dysbiosis in broilers and pigs under heat stress and its role in disrupting the gut barrier function

Several studies exist in the literature investigating the effect of heat stress on gut microbiota composition in broilers. In a study from Song et al. [78], heat stress decreased viable counts of *Lactobacillus* and increased viable counts of the opportunistic pathogen *Escherichia coli* in cecal contents in Ross 308 broilers subjected to cyclic heat stress (33 °C for 10 h/d) from 21 to 42 days of age. Heat stress was accompanied by a reduced performance, an impaired intestinal morphology and a decreased jejunal transepithelial electrical resistance in the broilers. Likewise, the same group reported in another study with Ross 308 broilers that cyclic heat stress (33 °C for 10 h/d) from 22 to 42 days of age led to lower viable counts of beneficial bacterial genera, such as *Lactobacillus* and *Bifidobacterium*, and increased viable counts of opportunistic pathogens, like *Coliforms* and *Clostridium*, in small intestinal digesta compared with broilers raised at thermoneutral temperature (22 °C) [44]. This study also revealed decreases of jejunal transepithelial electrical resistance and expression of tight junction proteins and an increase of jejunal paracellular permeability. Both studies strongly suggested that heat stress-induced gut microbiota alterations are accompanied by an impaired gut barrier function in broilers. In another study from Zhang et al. [28] it was demonstrated that broilers exposed to cyclic heat stress (33 °C for 10 h/d) from 21 to 42 days of age had lower abundances of *Lactobacillus* and *Bifidobacterium* and higher abundances of *Salmonella, E. coli* and *Clostridium* compared to age-matched broilers kept under thermoneutral conditions (22 °C). As expected, broilers’ performance and intestinal morphology were impaired and intestinal goblet cell number was reduced in heat stressed broilers of this study. In addition, this study revealed that jejunal expression of tight junction proteins (CLDN1, OCLN, ZO1, E-cadherin) and mucins (MUC2) were decreased, while parameters of intestinal permeability (serum *D*-lactic acid concentration and diamine oxidase activity) were elevated in heat stressed broilers indicating intestinal barrier disruption in broilers under heat stress. Changes in the gut microbiota composition were also shown in a study with Arbor Acres broilers exposed to cyclic heat stress (32 °C for 10 h/d) from 22 to 35 days of age [14]. However, the authors of this study reported alterations in the abundance of other bacterial genera; while the cecal abundances of *Parabacteroides*, *Saccharimonas*, *Romboutsia* and *Weissella* were increased, those of *Anaerofustis*, *Pseudonocardia*, *Rikenella* and *Tyzzerella* were decreased in the heat stressed broilers. Nevertheless, the cecal microbiota alterations in the heat stressed broilers were also accompanied by an impairment of intestinal morphology (decreased villus height and increased villus height-to-crypt depth ratio in duodenum and ileum), and an induction of inflammatory cell infiltration and lamina propria edema in the intestinal mucosa. In a study from Shi et al. [79], the cecal microbiota composition was also reported to be altered in broilers kept under chronic heat stress (34–38 °C) compared to thermoneutral temperature from 14 to 42 days of age. Significant heat stress-induced alterations of the microbiota composition of the broilers were observed at 21 and 28 days of age, such as increased abundances of Firmicutes phylum and of the opportunistic pathogen *Anaeroplasma* and decreased abundances of Bacteroidetes phylum and of some beneficial bacterial genera, like *Bacteroides* and *Dorea*. As expected, the heat stress caused a significant impairment of broilers performance (average daily gain, average daily feed intake, feed conversion) and increased serum concentrations of heat shock protein 70 (HSP70) and cortisol being indicative of a systemic stress response. In contrast, only one study revealed no significant alteration of the ileal microbiota composition in Arbor Acres broilers raised under chronic heat stress (31 °C) from 28 to 42 days of age compared to broilers raised at normal ambient temperature (21 °C) [80]. Since the broilers of this study were exposed to heat stress only from 28 days of age, one explanation for the lack of effect might be that the gut microbiota structure of broilers at this age is more stable than in younger broilers, thus, making the gut microbiota more resilient towards environmental factors. Collectively, the vast majority of studies clearly shows that the structure of the gut bacterial community is altered in broilers under heat stress. A repeated observation from different studies was that the abundances of beneficial commensal bacteria, such as *Lactobacillus* and *Bifidobacterium*, are decreasing, while those of opportunistic pathogens, such as *E. coli* and *Clostridium*, are increasing under heat stress. This indicates that gut dysbiosis contributes to gut barrier disruption in heat stressed broilers. In fact, commensal bacteria including *Lactobacillus* and *Bifidobacterium* are well-documented to strengthen the gut barrier, prevent colonization by pathogenic bacteria and stimulate the gut-associated immune system. These mechanisms provide efficient protection against pathogen invasion and infection.

Like in broilers, several studies in pigs demonstrated that heat stress modifies the gut microbiota composition. Le Sciellour et al. [13] were the first to demonstrate heat stress-induced modification of the fecal microbiota in 23-week-old growing pigs exposed to chronic heat stress at 29 °C for 3 weeks. This study showed that heat stressed pigs had a higher abundance of Firmicutes and a lower abundance of Bacteroidetes – the two most abundant phyla in the porcine gut. In addition, heat stressed pigs had higher abundances of the low-abundance phyla Spirochaetes, Fibrobacteres, Actinobacteria and Tenericutes than pigs kept at normal ambient temperature. Further heat stress-induced changes in the fecal microbiota composition of the pigs were an increased abundance of Lachnospiraceae and decreased abundances of Lactobacillaceae and its genus *Lactobacillus*. Considering that Lactobacillaceae play an important role in protecting intestinal mucosa integrity and strengthening the gut barrier, the decrease of Lactobacillaceae observed in this study is likely a critical hallmark of heat stress-induced gut dysbiosis and an important step of gut barrier disruption in pigs under heat stress conditions. The heat stress-induced changes of fecal microbiota composition were accompanied by a decreased performance of the pigs (body weight gain, feed intake, feed conversion ratio). Unlike in the study of Le Sciellour et al. [13] with growing pigs, no alterations of fecal microbiota composition were found at the phylum level in pregnant sows exposed to chronic heat stress (28–32 °C) from 85 d of gestation until farrowing [81]. However, some modifications of fecal microbiota composition in heat stressed gestating sows were observed at the genus level; namely, increased abundances of *Halomonas*, two Ruminococcaceae genera, *Eubacterium coprostanoligenes group* and *Coprococcus* and decreased abundances of *Streptococcus* and *Bacteroidales RF16 group*. At the species level, the abundance of *Halomonas* was increased, whereas the abundances of several *Prevotella* spp. were reduced in the heat stressed sows. These microbiota alterations observed at the species levels might be relevant to explain the impaired intestinal barrier integrity in the heat stressed sows, which was evident from elevated plasma concentrations of LPS, LPS binding protein and HSP70. In fact, *Halomonas* is known as an opportunistic pathogen [82], while *Prevotella* spp. are important contributors to intestinal barrier integrity by stimulating the production of butyrate [83]. Albeit the heat stress-induced gut microbiota changes obviously differ between gestating sows and growing pigs, the changes observed are indicative of gut dysbiosis which is known to promote gut barrier disruption.

Heat stress-induced modifications of the ileal and cecal microbiota were reported by Xia et al. [84]. This study demonstrated that short-term heat stress (33 °C for 72 h) reduces ileal abundances of *Flavonifractor, Thiomonas* and *Bifidobacterium* and cecal abundances of *Lactobacillus, Anaerovibrio, Prevotella* and *Succinivibrio*. In contrast, ileal abundances of *Lawsonia*, *Actinobacillus, Chlamydia* and some Lachnospiraceae genera and cecal abundances of *Staphylococcus*, *Frischella, Bacteroides*, *Akkermansia, Lachnoclostridium* and *Lachnospiraceae_UCG_004* were increased under heat stress. It has to be emphasized, that these gut microbiota changes in response to heat stress occurred independent of reduced feed intake, because confounding effects of a dissimilar nutrient intake were eliminated due to the use of an additional pair-fed control group. While *Lawsonia, Chlamydia, Staphylococcus* and *Bacteroides* are known as potential opportunistic pathogens, which have been related to gut dysbiosis in pigs [85–87], the genera *Lactobacillus*, *Bifidobacterium* and *Prevotella* are important contributors to intestinal barrier integrity and protection from intestinal epithelial damage. Thus, these findings from Xia et al. [84] suggest that the gut microbiota modifications observed under heat stress predisposes the pigs to intestinal barrier defects, endotoxemia and systemic inflammation. Consistent with these heat stress-induced gut microbiota changes, the heat stressed pigs exhibited the characteristic heat stress-associated intestinal barrier disruption and systemic inflammation. This was evident from a reduced ileal villus height, a decreased cecal expression of tight junction proteins, elevated plasma concentrations of intestinal permeability indicators and increased plasma concentrations of proinflammatory cytokines (IL1β, IL1 receptor type 1) in the heat stressed pigs. In contrast, plasma levels of T-helper (Th) 1 cytokines, such as IL12 and interferon γ (IFNγ), were found to be reduced in pigs exposed to heat stress, which is indicative of a shift from Th1 to Th2 – a known marker of immunosuppression. Based on this, the authors speculated that heat stress induces immunosuppression by shifting the Th1-to-Th2 ratio, thereby impairing immune homeostasis and increasing the susceptibility towards infectious pathogens. Similar effects with regard to an increase in the abundance of opportunistic pathogenic bacterial genera, such as *Streptococcus*, *Acinetobacter* and *Kurthia*, in the intestine of growing pigs (30 kg body weight) exposed to chronic heat stress (35 °C for 7 d) have been reported from Xiong et al. [88]. In this study, heat stress was accompanied by the characteristic morphological injuries in the small intestine of the pigs, such as villi tips desquamation and lamina propria exposing, and decreases of villus height and villus height-to-crypt depth ratios in the small intestine. At the molecular level, an up-regulation of TLR2, TLR4, TNFα, IL6, IL8, and p65-NF-κB subunit in small intestine of heat stressed pigs was observed indicating activation of mucosal immune response via TLR/NF-κB signaling pathway under heat stress. Interestingly, in another study from Xiong et al. [89], in which the effect of short-term heat stress (35 °C for 24 h) was explored in growing pigs, similar shifts in the gut microbial community (e.g. increases in the abundances of *Acinetobacter* and *Kurthia*) were observed in response to long-term heat stress indicating that heat stress exerts its effect on gut microbial composition very rapidly. Recently, Hu et al. [39] also provided evidence for the development of gut dysbiosis in young growing pigs exposed to chronic heat stress (34 °C) for 21 d. This was evident from decreases in the abundance of beneficial bacterial genera, such as *Lactobacillus* and unknown Ruminococcaceae, and increases in the abundances of opportunistic pathogens, such as Campylobacterales, Selenomonadales, Veillonellaceae, Lachnospiraceae and Megasphaera, in the colon of the heat stressed pigs. The heat stress-induced gut dysbiosis also coincided with typical signs of intestinal mucosal impairment, such as decreased crypt depth, reduced number of mucosal goblet cells, and an increased histology score. In addition, diarrhea index and LPS concentrations in peripheral blood were elevated in heat stressed pigs. A striking aspect of this study was that fecal microbiota transplantation from heat stressed pigs to pseudo germ-free mice causes signs of intestinal inflammation and damage, such as lymphocyte infiltration in the colonic mucosa, decreases of colonic mucosal height and muscle layer thickness, and an increased histology score compared to mice receiving fecal microbiota from pigs kept under normal ambient temperature. The observation from this study that the detrimental effect of gut dysbiosis to colonic mucosa integrity is transmissible strongly suggests that the heat stress-induced gut dysbiosis plays a key role in impairing intestinal mucosa integrity. Given this context, strategies that counteract gut dysbiosis are decisive to prevent heat stress-induced impairment of gut integrity and function. Based on similar observations in both pigs and broilers, that heat stress causes a decrease in the abundances of beneficial commensal bacteria, such as *Lactobacillus* and *Bifidobacterium*, dietary supplementation with either probiotics (*Lactobacillus* spp., *Bifidobacterium* spp.) or prebiotics, which promote the growth of *Lactobacillus* and *Bifidobacterium*, might be an effective strategy to combat heat stress-induced gut dysbiosis and intestinal barrier defects.

### Role of gut dysbiosis in the reduction of feed intake under heat stress conditions

Apart from disrupting the gut barrier, heat stress-induced gut dysbiosis is likely also involved in feed intake reduction. The latter is one of the key characteristics of heat stress which significantly contributes to the impairment of animal’s growth performance under heat stress due to an inadequate intake of nutrients and energy required for growth. Regulation of feed intake is one of the key tasks of the hypothalamus, where peripheral feedback signals about the animal’s nutritional and metabolic status are sensed and integrated [90]. These peripheral signals comprise different peptide hormones secreted from the intestine (called incretins), such as peptide YY, glucagon-like peptide 1 (GLP-1) and ghrelin [91, 92], the pancreas, such as insulin and glucagon, and white adipose tissue, such as the energy storage feedback signal leptin [93]. Leptin is also a negative regulator of the endocannabinoid system (ECS), which represents a signaling system specifically facilitating energy storage through stimulating appetite and feed intake [94]. In the arcuatus nucleus of the hypothalamus two functionally antagonistic neuronal populations – one acting anorexigenic (appetite-lowering) and the other orexigenic (appetite-stimulating) – are present, which are targeted by the abovementioned peripheral signals and express and secrete either appetite-lowering [POMC, CART, α-melanocyte-stimulating hormone (αMSH)] or appetite-stimulating signals (AgRP, NPY) [90]. Through this regulatory system, the animal is capable of tightly adapting feed intake to the body’s demand. Several studies in avian species including broilers have demonstrated an altered expression of hypothalamic neuropeptides or peripheral signals involved in feed intake regulation, such as NPY and ghrelin, under conditions of heat stress [71–74] indicating that heat stress affects feed intake regulation.

Recent evidence clearly shows that the gut microbiota affects feed intake regulation of the host through various microbiota-derived signals, such as SCFA, bile acids and MAMP like LPS. These signals act as signaling molecules between microbes and the host along the gut-liver axis. Thus, it is likely that the heat stress-induced modification of the gut microbiota composition in broilers and pigs contributes to feed intake reduction under heat stress. As already described above, an increased translocation of MAMP including LPS as a consequence of gut dysbiosis causes induction of hepatic and hypothalamic inflammation [65]. Hypothalamic inflammation results in activation of the hypothalamic neuronal populations that produce anorexigenic neuropeptides, while inhibiting those that produce orexigenic neuropeptides, thereby, reducing appetite and feed intake [66]. This mechanism explains why intravenous administration of LPS and proinflammatory cytokines, which cause hypothalamic inflammation, to livestock animals including growing pigs strongly reduces feed intake [68, 69]. Besides MAMP, gut microbiota-derived SCFA are also well-documented to affect animal’s feed intake regulation through stimulating the peripheral secretion of satiety-inducing leptin [95], but also by affecting secretion of incretins, like peptide YY and GLP-1 [96]. Interestingly, studies have shown that the contribution of individual SCFA to feed intake regulation is different; e.g. butyrate and to a lesser extent propionate, but not acetate stimulate the secretion of peptide YY and GLP-1 [96]. Since changes in the gut microbiota composition lead to changes in the amount and profile of SCFA produced from microbial fermentation, as also demonstrated in pigs subjected to heat stress [81, 84, 89, 97], it is highly likely that heat stress-induced gut microbiota alterations affect feed intake regulation via SCFA.

To the best of our knowledge, the effect of heat stress on the animal’s ECS has not been investigated so far. This is at least slightly surprising considering that the ECS plays a central role in regulating animal’s feed intake and the central ECS is under negative control by the peripheral energy storage feedback signal leptin. In addition, feed intake was clearly shown to be regulated by the gut microbiota through modulating the intestinal ECS [98]. Muccioli et al. [98] demonstrated not only that the gut microbiota regulates important parts of the intestinal ECS (e.g. intestinal levels of anandamide and cannabinoid receptor type 1 mRNA), but also that obesity-induced dysregulation of the peripheral ECS in the intestine causes a disruption of the intestinal barrier, which is also a critical event during heat stress. Thus, future studies should focus on the effect of heat stress on the animal’s central and peripheral ECS. This clearly shows that heat stress-induced changes in the gut microbiota composition might affect animal’s appetite and feeding behavior through the gut-liver axis. Interestingly, in the abovementioned study from Xiong et al. [88] correlations were found between feed intake and specific intestinal bacterial groups in growing pigs exposed to heat stress. According to this study, feed intake is negatively correlated with the opportunistic pathogenic genera *Acinetobacter* and *Kurthia*, whose abundances in the intestine were increased by both acute and chronic heat stress [88, 89], but positively correlated with the abundance of *Lactobacillus* genus and *Lactobacillus* spp., such as *Lactobacillus reuteri*. These findings suggest that modifying the gut microbiota composition under heat stress by supplementation of *Lactobacillus* spp. probiotics or prebiotics, which promotes the growth of *Lactobacillus* spp. and inhibits the growth of opportunistic pathogens, might be a reasonable approach to stimulate feed intake and to alleviate the heat stress-induced impairment of gut function and animal performance.

## Potential of dietary probiotics, prebiotics and synbiotics to restore the function of the gut-liver axis in heat stressed monogastric farm animals

As described above, heat stress-induced gut dysbiosis plays a key role in impairing intestinal mucosa integrity and developing systemic and hypothalamic inflammation, which are critical events underlying the impairment of farm animal’s productivity, health and welfare under heat stress. Thus, strategies that counteract gut dysbiosis are decisive to alleviate the detrimental impact of heat stress on farm animals. Since a “healthy” gut microbiota composition is vital for protection against intestinal barrier disruption, dietary concepts aiming to restore a healthy gut microbiota structure likely protect animals from heat stress-induced impairment of gut-liver axis function. This aim is followed particularly by dietary probiotics, prebiotics and synbiotics, the latter referring to the combination of probiotics and prebiotics with the aim of enabling additive and synergistic effects of the two bioactive feed additives. The beneficial effect of probiotics, most of which belong to the genera *Lactobacillus* (*L. acidophilus*, *L. rhamnosus*, *L. plantarum*, *L. reuteri*), *Bifidobacterium* (*B. infantis*, *B. bifidum*, *B. animalis*), *Bacillus* (*B. licheniformis*, *B. subtilis*) and *Enterococcus* (*E. faecium*, *E. faecalis*) on gut homeostasis and gut integrity can be attributed to various mechanisms, such as promotion of the growth of beneficial commensal bacteria, inhibition of the growth of enteric and opportunistic pathogens, degradation of bacterial antigens, strengthening of the gut barrier, inhibition of inflammatory processes and stimulation of gut immunity [99]. Prebiotics include various indigestible carbohydrates, such as cello-oligosaccharides (COS), fructo-oligosaccharides (FOS), galacto-oligosaccharides (GOS) and mannan-oligosaccharides (MOS), which serve as growth substrates for specific bacterial groups including the abovementioned probiotic bacterial genera which are beneficial for host’s health. Since prebiotics promote the growth of probiotic bacteria, it is not surprising that prebiotics have similar effects on the gut-liver axis as the probiotics, whose growth is promoted, and also contribute to gut homeostasis and gut integrity.

### Evidence from studies in broilers exposed to heat stress

Regarding the efficacy of probiotics in broilers exposed to heat stress several studies have been conducted. A study with Ross 308 broilers clearly showed that a probiotic mixture (*B. licheniformis*, *B. subtilis*, *L. plantarum*) is effective in ameliorating heat stress-induced impairment of growth performance, gut barrier integrity (decreased expression of the tight junction proteins) and gut morphology (reduced jejunal villus height) [44]. In addition, the probiotic mixture was able to counteract some of the alterations in the abundance of selected gut microbial populations as induced by the heat stress regimen applied (33 °C for 10 h/d from 22 to 42 days of age); remarkably, the heat stress-induced decrease in the viable counts of *Lactobacillus* and *Bifidobacterium* and increase of *Coliforms* was completely alleviated by the probiotic mixture suggesting that probiotics are able to prevent heat stress-induced gut dysbiosis in broilers. In another study, dietary supplementation of the probiotic *B. subtilis* from 1 to 43 days of age to Ross 708 broilers exposed to heat stress from 15 to 43 days of age (32 °C for 10 h/d) clearly improved broilers performance [100]. The positive effect of the probiotic was accompanied by an alleviation of heat stress-induced elevation of IgA and IgY concentrations in cecal tonsils and of heat stress-induced expression of proinflammatory and stress-responsive genes in the liver, which indicates an inhibition of heat stress-induced intestinal and systemic inflammation, and a partial restoration of the heat stress-induced impairment of gut-liver axis. In addition, supplementation of the probiotic was found to cause several behavioral changes of the heat stressed broilers being indicative of an improved animal health and welfare, i.e., the supplemented heat stressed broilers spent more time with standing and walking and less time with sitting, sleeping and dozing and showed less heat-associated behaviors, such as panting, wing spreading and squatting close to the ground. In addition, the broilers supplemented with the probiotic spent less time with drinking, but more time with foraging and eating under heat stress. Due to the fundamental role of an intact gut-liver axis for animal health, it is likely that the partial restoration of the heat stress-induced impairment of gut-liver axis by the probiotic in the study from Wang et al. [100] has contributed to the behavioral changes of the broilers enabling them to better cope with heat stress. A further study with Arbor Acres broilers demonstrated, that dietary supplementation of a probiotic mixture (*L. acidophilus*, *L. plantarum*, *E. faecalis*) alleviates the negative effects of chronic heat stress (35 °C for 12 h/d from 22 to 42 days of age), such as reductions of villus height and villus height-to-crypt depth ratio in intestinal mucosa and increases of serum levels of LPS, TNFα and IL6 [101]. This observation also represents convincing evidence of the efficacy of probiotic supplementation in protecting heat stressed broilers from impairment of the gut-liver axis function. Moreover, Abdelqader et al. [102] demonstrated that dietary supplementation of the probiotic *Bacillus subtilis* attenuates the detrimental effect of chronic heat stress (30 °C for 24 h/d from 22 to 35 days of age) on performance (reduced body weight gain, decreased feed efficiency), intestinal microarchitecture (reduced villus height, crypt depth, villus surface area and absorptive epithelial cell area) and microbiota composition in Ross 308 broilers.

Regarding the efficacy of prebiotics, a study with 15-day-old Ross broilers revealed that dietary GOS strongly decreased induction of jejunal genes involved in gut barrier integrity and inflammation (HSP70, HSP90, CLDN5, ZO1, TLR4, IL6, IL8) in response to cyclic heat stress (38–39 °C for 8 h/d on 5 consecutive days) [103]. This indicated that dietary prebiotics inhibit the effect of heat stress on stress-responsive gene expression in small intestine. Whether or not the supplementation of GOS was efficacious in preventing intestinal barrier disruption in broilers exposed to heat stress cannot be answered from this study due to lack of data. In another study, the effect of COS was investigated in 21-day-old Ross broilers that were exposed to cyclic heat stress (33 °C for 10 h/d) until a broilers age of 42 d [78]. Heat stress reduced performance parameters, impaired intestinal morphology in the jejunum (villus height, villus height-to-crypt depth ratio), increased intestinal paracellular permeability, decreased cecal viable counts of *Lactobacillus* and increased cecal viable counts of *E. coli* and *Clostridium* as compared to broilers kept at normal ambient temperature. In contrast, supplemental COS enhanced growth performance, improved intestinal morphology, decreased jejunal paracellular permeability, increased cecal viable counts of *Lactobacillus* and decreased cecal viable counts of *E. coli* and *Clostridium* in the broilers under heat stress when compared to non-supplemented control broilers. This indicated that supplementation with a COS prebiotic partially ameliorates the adverse effects of heat stress in broilers through improving gut-liver axis function.

In line with the above studies showing a positive effect of probiotics and prebiotics on gut-liver axis function in broilers under heat stress, a study with dietary supplementation of a synbiotic consisting of a mixture of probiotic strains (*B. animalis*, *E. faecium*, *L. reuteri*, *Pediococcus acidilactici*) and FOS had a beneficial impact on cecal microbiota composition [decreased viable counts of detrimental bacteria (*E. coli*, *Coliforms*) and increased viable counts of beneficial bacteria (*Bifidobacterium* spp., *Lactobacillus* spp.)] in Ross 708 broilers exposed to cyclic heat stress (32 °C for 9 h/d) from 15 to 42 days of age [104]. The modification of the gut microbiota composition by the synbiotic was accompanied by an improved performance of the broilers and a stimulatory effect on the immune system, as evidenced from an increased number of blood lymphocytes and a reduced blood heterophils-to-lymphocytes ratio [104]. In contrast, in a study of Sohail et al. [105] dietary supplementation with a synbiotic consisting of a probiotic mixture (*L. plantarum*, *L. delbrueckii* ssp. Bulgaricus, *L. acidophilus*, *L. rhamnosus*, *B. bifidum*, *Streptococcus salivarius* ssp. Thermophilus and *E. faecium*) and MOS in Ross 708 broilers did not ameliorate the chronic heat stress (35 °C from 1 to 42 days of age)-induced impairment of body weight gain, but supplementation with the prebiotic alone did. Despite the lack of effect of the synbiotic on growth performance of heat stressed broilers in this study, the synbiotic was able to partially attenuate the heat stress-induced impairment of ileal villus width, surface area and crypt depth indicating a beneficial effect of the synbiotic on the small intestinal microarchitecture. However, in another study of this group, dietary supplementation of a similar synbiotic (probiotic mixture and MOS) was demonstrated to be efficacious in improving the intestinal microarchitecture (villus height, villus width, crypt depth, villus height-to-crypt depth ratio) in broilers exposed to cyclic heat stress (35 °C for 8 h/d) from 22 to 42 days of age [106]. In addition, the synbiotic increased the number of mucus-producing goblet cells in small intestine of the heat stressed broilers suggesting that the synbiotic is able to strengthen the local mucosal barrier function under heat stress.

A further promising feeding concept to reduce the negative impact of heat stress in broilers might be the use of postbiotics, also called paraprobiotics. The term postbiotics refers to the metabolites and bioactive compounds produced from live probiotic strains which have been inactivated by different methods (e.g. heat, ultraviolet light, chemical treatment). At least a few studies from the same group in broilers demonstrated that dietary supplementation of postbiotics (produced from different *L. plantarum* strains) mitigates the adverse impact of heat stress on growth performance, gut microbiota composition, gut barrier function, stress response and meat quality [107–109]. However, more studies from independent groups are necessary to provide sufficient evidence for the efficacy of this concept in broilers.

### Evidence from studies in pigs exposed to heat stress

**Fig. 3:**
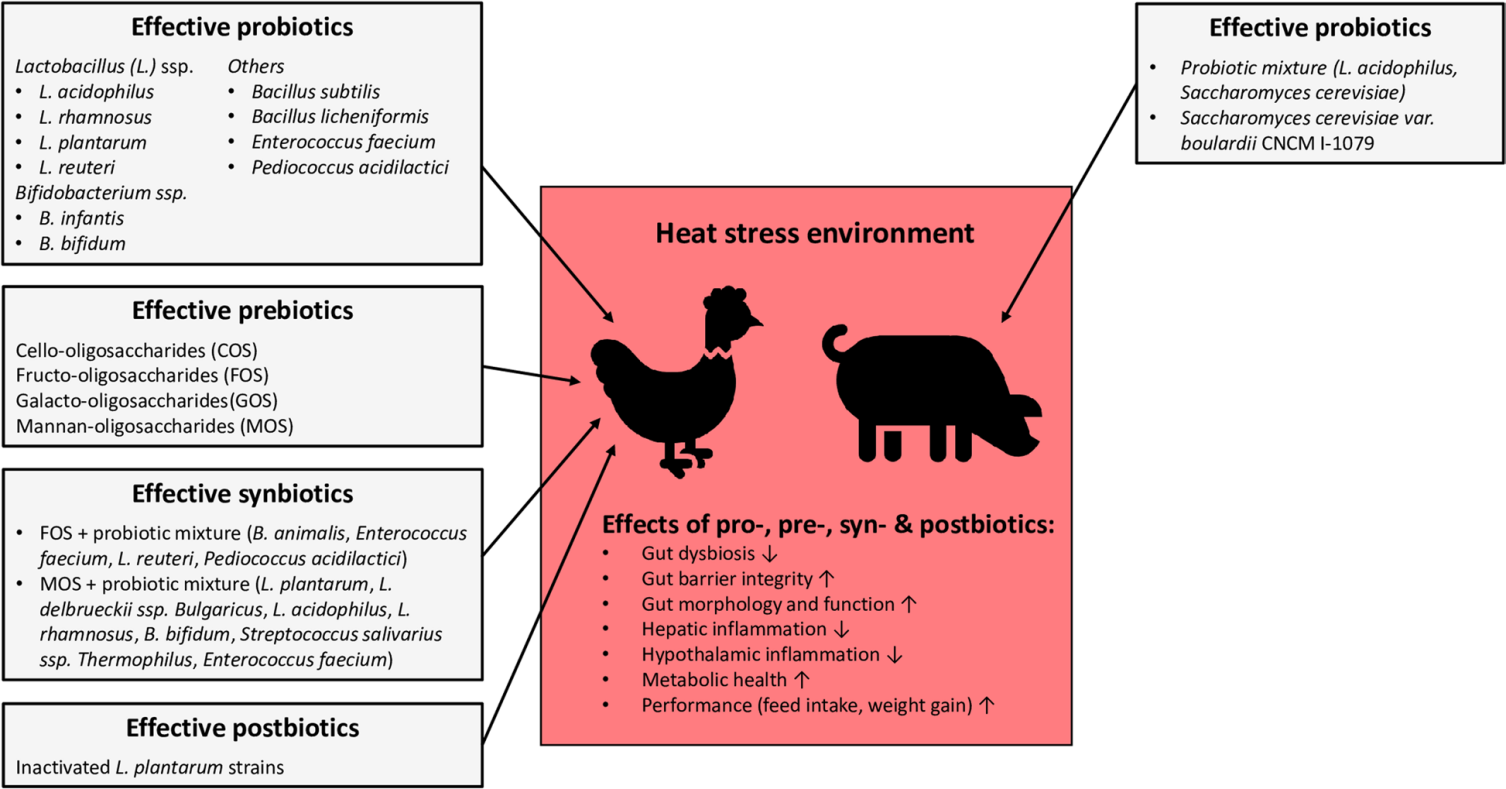
Efficacy of dietary probiotics, prebiotics, synbiotics and postbiotics to restore the gut-liver axis function, metabolic health and performance in broilers and pigs exposed to heat stress. Convincing evidence exists that feeding of probiotics (mainly *Lactobacillus (L.)* ssp. and *Bifidobacterium (B.)* ssp.), prebiotics (cello-, fructo-, galacto- and mannan-oligosaccharides) and synbiotics (different probiotic mixtures with either cello- or galacto-oligosaccharides) is an efficacious strategy to protect broilers from heat stress-induced gut dysbiosis, impairment of gut barrier integrity and gut morphology and function, hepatic inflammation, hypothalamic inflammation, impairment of metabolic health and reduction of performance. In addition, results from a few studies show that dietary postbiotics based on inactivated *L. plantarum* strains alleviate the adverse impact of heat stress in broilers. In pigs, only a few studies demonstrated that feeding of probiotics (probiotic mixtures or a specific *Saccharomyces cerevisiae* strain) is also a suitable approach to protect from heat stress-induced impairment of gut integrity and function, metabolic health and performance. However, no studies are available in pigs investigating the efficacy of prebiotics, synbiotics and postbiotics on gut health, metabolic health and productivity under heat stress

In contrast to broilers, only very few studies investigated the effect of probiotics on heat stress-induced impairment of the function of the gut-liver axis. In a study from Gan et al. [110], supplementation of a probiotic mixture (*L. acidophilus* and *Saccharomyces cerevisiae*) alone or in combination with selenium downregulated stress-responsive genes (HSP27, HSP70) in liver, kidney and spleen of 4-week-old piglets kept under chronic heat stress for up to 42 d, whereby a stronger effect has been observed with the selenium-enriched probiotic. This indicated that probiotics are effective in reducing heat stress-induced systemic stress response in pigs, likely due to alleviating the negative impact of heat stress on gut barrier function. Unfortunately, gut microbiota composition and performance data were not reported in this study. In a recent study from Labussière et al. [111], the effect of supplementation of a live yeast (*Saccharomyces cerevisiae var. boulardii* CNCM I-1079) on feeding behavior, energy metabolism and fecal microbiota composition was investigated in finishing boars exposed to chronic heat stress (28 °C) for 13 d. This study showed that the dietary probiotic increased feed intake, the number of meals and energy retention during heat stress. In addition, the skin temperature during heat stress increased to a lesser extent in the pigs supplemented with the probiotic than in the non-supplemented pigs indicating better adaptation to heat stress in response to probiotic supplementation. Fecal microbiota analysis revealed that the supplementation with the probiotic decreased the abundance of *Ruminoccocus*, *Coprococcus*, *Peptococcus* and *Oxalobacter* genera and increased the abundance of some beneficial bacterial genera, such as *Lactococcus* and *Subdoligranulum*, during heat stress. Amongst *Lactococcus* spp. *L. lactis* is known to produce nisin, a bacteriocin that likely relieves the host immune system. Given that the immune response is associated with the utilization of energy substrates, the authors postulated that the relieve of the immune system possibly allows some energy saving by the host. This observation may explain that *L. lactis* abundance was positively correlated with the energy retention-to-metabolizable energy ratio and dry matter intake in the finishing boars under heat stress [111]. Based on their results, the authors suggested that dietary probiotic supplementation enables the pigs to better cope with heat stress through a beneficial modulation of the microbiota. To the best of our knowledge, no studies are available in pigs investigating the efficacy of prebiotics, synbiotics and postbiotics on performance and intestinal and animal health under heat stress. Given the beneficial effects of prebiotics, synbiotics and postbiotics on production features and intestinal health in broilers exposed to heat stress, future studies have to show whether this feeding strategy is also efficacious in growing pigs, growing-finishing pigs and sows. In Fig. 3, the efficacy of dietary strategies using probiotics, prebiotics, synbiotics and postbiotics to reduce the adverse impact of heat stress in pigs and broilers is comparatively summarized.

## Conclusions and perspectives

Convincing evidence in the literature exists that gut dysbiosis, a term used to describe a perturbation of commensal gut microbiota, develops in broilers and pigs under heat stress. Owing to the protective role of commensal bacteria for the gut barrier, gut dysbiosis causes a disruption of the gut barrier leading to endotoxemia, which contributes to the typical characteristics of heat stressed broilers and growing and growing-finishing pigs, such as reduced feed intake, decreased growth and reduced lean carcass weight. A substantial number of studies show that feeding of probiotics, prebiotics and synbiotics is an efficacious strategy to protect broilers from heat stress-induced gut barrier disruption through altering the gut microbiota (increase in the number of beneficial bacterial groups, decrease in the number of opportunistic pathogens) and promoting all decisive structural (e.g. tight junctions), biochemical (e.g. mucins, antimicrobial peptides), and immunological elements (e.g. secretory IgA) of the intestinal barrier. In most of the available studies in heat stressed broilers, the alterations of gut microbiota and improvements of gut barrier function induced by feeding of either probiotics, prebiotics or synbiotics were accompanied by an improved productivity (increased body weight gain, enhanced feed intake, elevated feed conversion), health and/or welfare when compared to non-supplemented broilers exposed to heat stress. These findings indicate that the restoration of gut homeostasis and function is a key target for dietary interventions aiming to provide at least partial protection of broilers from the detrimental impact of heat stress conditions. Despite that the number of studies dealing with the same feeding strategy in heat stressed pigs is limited, the few studies available suggest that feeding of probiotics could also be a suitable approach to enhance productivity, health and welfare in pigs kept under heat stress conditions. However, substantially more studies with pigs are necessary to reliably judge the efficacy of the probiotic feeding concept in improving growth, health and welfare of this farm animal species. While the effectiveness of prebiotics and synbiotics in improving production features and intestinal health of broilers exposed to heat stress has been proven, this feeding strategy has not been investigated in pigs under heat stress. Thus, future studies have to show whether prebiotics and synbiotics are also efficacious in improving productivity, health and welfare of pigs exposed to heat stress conditions. Given that a few studies from the same group demonstrated that dietary supplementation of postbiotics alleviates the adverse impact of heat stress in broilers, feeding of postbiotics might be a further suitable strategy to reduce the detrimental impact of heat stress in broilers. However, more studies from independent groups are necessary to substantiate these findings in broilers. Due to the complete lack of studies dealing with dietary postbiotics in pigs under heat stress, future studies are warranted to test the efficacy of this feeding concept in pigs. The implementation of dietary postbiotics as a feeding concept to reduce the detrimental impact of heat stress on productivity, health and welfare in broilers and pigs (in the case of effectiveness) might be promising, because the use of postbiotics in the feeding of farm animals is advantageous compared to probiotics due to the long shelf life and the lack of necessity for cooling during transportation and storage.

## Data Availability

Not applicable.

## Abbreviations

AgRP: Agouti-related peptide
αMSH: α-Melanocyte-stimulating hormone
CART: Cocaine- and amphetamine-regulated transcript
CLDN: Claudin
COS: Cello-oligosaccharides
ECS: Endocannabinoid system
FOS: Fructo-oligosaccharides
GLP-1: Glucagon-like peptide 1
GOS: Galacto-oligosaccharides
HSP: Heat shock protein
IEC: Intestinal epithelial cell
IFNγ: Interferon γ
IgA: Immunoglobulin A
IL: Interleukin
LPS: Lipopolysaccharide
MAMP: Microbial-associated molecular patterns
MOS: Mannan-oligosaccharides
MUC: Mucin
MyD88: Myeloid differentiation primary response 88
NF-κB: Nuclear factor-κB
NPY: Neuropeptide Y
OCLN: Occludin
POMC: Proopiomelanocortin
PRR: Pathogen recognition receptor
SCFA: Short-chain fatty acid
Th: T-helper
TLR: Toll-like receptor
TNFα: Tumor necrosis factor α
ZO: Zonula occludens
